# 2009 ACMP Meeting – Young Investigators' Sympympymposium Abstracacts

**DOI:** 10.1120/jacmp.v10i3.3106

**Published:** 2009-07-21

**MO‐F‐Sandler‐01**

**Effects of breathing variation on internal target volume (ITV) in respiratory gated radiation therapy**

J Cai^a^

R McLawhorn

K Sheng

P Read

J Larner

S Benedict

*University of Virginia, Charlottesville, VA, USA.*

**Purpose**

To investigate the effects of breathing variation on ITV in respiratory‐gated radiation therapy.

**Method and Materials**

7 volunteers and 5 lung cancer patients underwent a 5‐minute MRI scan in the sagittal plane to acquire dynamic MRI (dMRI) of lung motion, from which motion trajectories of the tumor (for patients) or a pulmonary vessel (for volunteers) were determined. A MATLAB program was written to simulate cine‐mode 4DCT by segmenting/resorting dMRI. Image motion phantoms were created by moving a round‐disk (mimicking tumor) with dMRI‐determined trajectories for all subjects. Simulated 4DCT (sCT) were generated from phantom images, and also from dMRI for patients. Internal target area (ITA, 2D counterpart of ITV) in the gated window was determined from both sCT and dMRI in each of the phantom and patient studies, from which the area $\left({\text{ITA}}_{\text{GW}}\right)$, major axis (*L1*) and minor axis (*L2*) were determined and compared. Similarity between the two ITAs was also calculated.

**Results**

In the phantom study with a 3 cm “tumor”, measurements in dMRI are all significantly (p‐value<0.001) greater than those in sCT (${\text{ITA}}_{\text{GW}}=992.9\pm 121.7\;{\text{mm}}^{2},\;L1=38.1\pm 3.8\;\text{mm},\;L2=33.1\pm 1.0\;\text{mm}$ in $\text{dMRI};\;{\text{ITA}}_{\text{GW}}=836.7\pm 66.6\;{\text{mm}}^{2},\;L1=34.1\pm 2.4\;\text{mm},\;L2=31.2\pm 0.7\;\text{mm}$ in sCT). These differences are even greater with a 1 cm “tumor”. Similar results were found in the patient study (${\text{ITA}}_{\text{GW}}=1554.4\pm 822.5\;{\text{mm}}^{2},\;L1=49.9\pm 11.3\;\text{mm},\;L2=38.5\pm 11.9\;\text{mm}$ in $\text{dMRI};\;{\text{ITA}}_{\text{GW}}=1319.0\pm 852.7\;{\text{mm}}^{2},\;L1=43.2\pm 14.0\;\text{mm},\;L2=36.8\pm 12.0\;\text{mm}$ in sCT). Similarity between two ITAs is 0.79±0.10 and 0.83±0.09 in the phantom study and the patient study, respectively.

**Conclusion**

4DCT may underestimate the gated window ITV. An additional margin to account for the breathing variation may be necessary in determining gated window PTV.

**MO‐F‐Sandler‐02**

**Investigation of the dosimetric consequences based on imaging used for conventional, gated, and tracking radiotherapy of mobile tumors**

Teboh Roland^a^

Y Liu

N Papanikolaou

*The University of Texas Health Science Center at San Antonio, San Antonio, TX, USA.*

**Purpose**

Investigate the dosimetric consequences based on imaging used for conventional, gated, and tracking radiotherapy of mobile tumors.

**Method and Materials**

Data from two patients previously treated for lung cancer with approximately 1 cm tumor extent of motion was used. Both 3D and 4D dose distributions were computed, with 4D accounting for organ motion and based on a 4DCT image set while 3D dose was based on a static anatomy. The 4D dose was derived from multiple 3D plans corresponding to various phases of the respiratory cycle via a validated non‐rigid deformable image registration algorithm. We compared the 3D versus 4D plan predicted lung volume irradiated by at least 20Gy (V20), mean lung dose (MLD), isocenter point dose (IPD), and the target generalized equivalent uniform dose (GTV‐gEUD and PTV‐gEUD) for conformal radiotherapy (no margins to account for motion – 3DCRT; with 4DCT derived margins to account for motion – 4D Static), gated, and tracking radiotherapy.

**Results**

The discrepancies between the 3D and 4D plan predictions were: 17%, 4%, 1%, 1% for lung V20; 8%, 7%, 2%, 2% for the PTV‐gEUD; 1%, 4%, 0.6%, 2% for MLD; 0.6%, 2%, 0.8%, 0.4% for the GTV‐gEUD, and 0.2%, 0.7%, 0.3%, 0.7% for the IPD for the delivery techniques 3DCRT, tracking, 4D static, and gating, respectively.

**Conclusion**

Although we observed some trend in the dosimetric parameters considered (for example, the discrepancies across all parameters for the 4D static and gating techniques were within 2%), further studies involving varied tumor characteristics are required for any concrete conclusions.

**MO‐F‐Sandler‐03**

**Intensity‐modulated proton planning for ocular tumor using human anatomy dose algorithm and preliminary comparison with IMRT planning**

B Massingill^a^

Y Liu

A Diaz

N Papanikolaou

C Esquivel

*Cancer Therapy Research Center, Radiation Oncology, University of Texas Health Science Center at San Antonio, San Antonio, TX, USA.*

**Purpose**

We aim to provide accurate dose calculations for ocular tumor and adjacent critical organs using intensity‐modulated proton therapy (IMPT) using a human anatomy‐based Monte Carlo model. Dose is simulated using the Monte Carlo code MCNPX and compared to standard photon IMRT planning using Pinnacle3 TPS.

**Method and Materials**

The human anatomy model was adapted from the Visible Human Project from the National Library in Medicine. Sectioned images were assigned physical properties. Two independent trials were developed using IMRT and IMPT, respectively. Isodose lines and dose profiles for each transverse, sagittal, and coronal view of the VHP model were provided for planning evaluation. Dose volume histogram in eyes, optic nerves, brain, chiasm, lacrimal, pituitary, lens, and PTV were compared between IMRT and IMPT.

**Results**

The ocular tumor was well covered by 95% in IMRT and 70% isodose in IMPT. Comparing the IMRT and IMPT, the mean dose was 4508 cGy and 3762 cGy‐Eq for PTV, 2770 cGy and 1524 cGy‐Eq for the eye, 3300 cGy and 1192 cGy‐Eq for lens, 794 cGy and 162 cGy‐Eq for optic nerve, 193 cGy and 20 cGy‐Eq for lacrimal, 26 cGy and 0.0 cGy‐Eq for brain, 120 cGy and 0.0 cGy‐Eq for chiasm, 272 cGy and 0.00 cGy‐Eq for pituitary, respectively.

**Conclusion**

IMPT provided conformal dose to the ocular tumor and significantly spared dose to the surrounding critical organs compared to IMRT. Human anatomy‐based Monte Carlo dose potentially provides more accurate dose calculations when accounting for the tissue component in the eyes.

**MO‐F‐SANDLER‐04**

**Radiobiologie evaluations of stereotactic body radiation therapy (SBRT) treatment plans in patients with non‐small‐cell lung cancer (NSCLC)**

T De La Fuente Herman^1,a^

T Herman^1^

K Hibbitts^1^

M Vlachaki^2^

J Stoner^1^

S Ahmad^1^

*The University of Oklahoma Health Sciences Center,^1^ Oklahoma City, OK, USA; Wayne State University,^2^ Detroit, MI, USA.*

**Purpose**

To evaluate radiobiological effective doses from SBRT treatment plans.

**Materials and Methods**

20 patients with stage I NSCLC were scanned using 4DCT techniques. Treatment planning was performed with 6 MV non‐opposing coplanar beams using Eclipse treatment planning system with tissue heterogeneity corrections. Prescription dose was 60 Gy in three fractions. Based on the universal survival curve (USC),^(1)^ the biologically effective dose (BED), standard effective dose (SED), and single fraction equivalent dose (SFED) values were calculated:

$$BED=\frac{1}{\alpha \cdot D_{0}}(D-n\cdot D_{q})\;\;;\;\;SFED=D-(n-1)\cdot D_{q}\;\;;\;\;SED=\frac{BED}{1+\frac{2}{\left(\alpha /\beta \right)}}$$

Dose volume histograms (DVH) were used to generate equivalent uniform doses (EUDs) calculated with donogen cell $\text{density}=220\;\text{million},\;{\text{SF}}_{2}=0.4$, and α/β=10, or 8.605 (Park et al). EUDs replaced *D* in the equations above.

**Results**

The minimum, mean and maximum PTV doses were 53.07, 62.23 and 66.32 Gy, respectively. The dose heterogeneity index $\left(D_{1\%}/D_{99\%}\right)$ was 1.14%. Accounting for heterogeneities from tissue and dose distributions, the SFED was 4% less than Park's value.

**Conclusions**

The SFED^(1)^ with EUD is suitable for describing and comparing SBRT prescription schemes as it incorporates the strengths of the linear quadratic model for doses near the shoulder region and the multi‐target model for large doses that fall on the exponential part of the cell survival curve, giving an accurate description of the equivalent potency of SBRT. Use of EUD is more biologically relevant than use of prescribed dose, since it falls between minimum and mean dose on the DVH.

1. Park et al.: Int J Radiat Oncol Biol Phys. 2008;70:847–852

**MO‐F‐Sandler‐05**

Setup Margins Requirements for Stereotactic Lung Radiotherapy^*^

T Niedermayr^a^

M Herman

*Mayo Clinic, Rochester, MN, USA.*

**Purpose**

Optimum treatment efficacy requires that setup uncertainties be determined for each institution, treatment site, and technique. This work investigates how the use of volumetric image guidance affects tumor targeting accuracy and tumor volume margin requirements in SBRT lung patients. These results are compared to the RTOG 0618 SBRT lung protocol requirements.

**Method and Materials**

Setup uncertainties and margins were established for forty‐five patients treated with stereotactic lung radiotherapy using the Varian OBI CBCT imager for daily target localization. At each treatment fraction, at least three CBCTs were acquired to: 1) assess the initial required shifts, 2) verify the patient position before treatment, and 3) assess the patient position after treatment.

**Results**

The group margins were calculated according to the Van Herk formalism with the intent to give 95% of the prescribed dose to 90% of the patient population. With laser alignment, an average margin of 20.6 mm in each direction is required. After CBCT localization, the average margin requirement in each direction is reduced to 3.3 mm. If the intrafraction tumor motion is taken into account, this margin is increased to 4.0 mm.

**Conclusion**

The amplitude of the 95%–90% margin (3–5 mm, depending on direction) is consistent with the RTOG requirements of 5–10 mm. Larger margins might be required if higher dose or population coverages are intended.

^*^Conflict of Interest: This research was funded in part by Varian Medical Systems.

